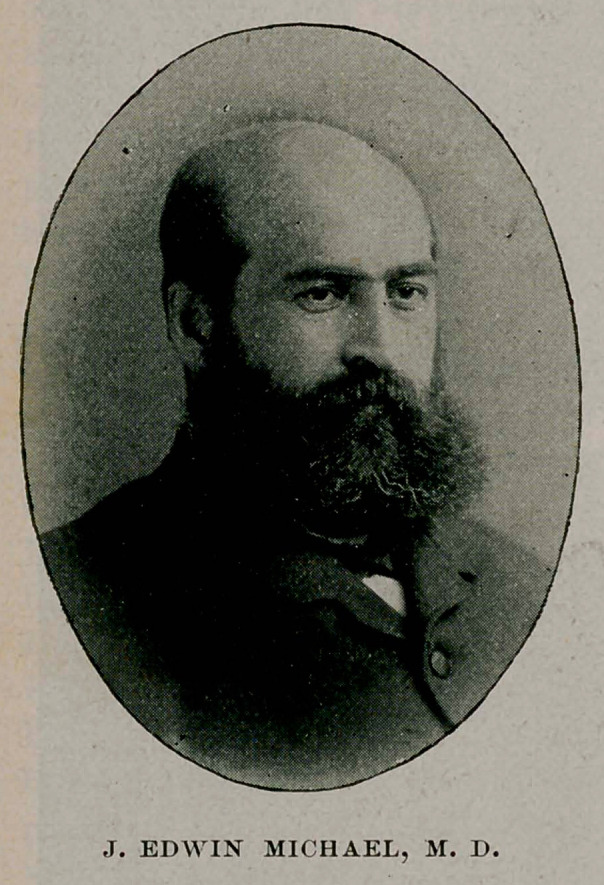# Dr. J. Edwin Michael

**Published:** 1896-01

**Authors:** 


DR. J. EDWIN MICHAEL, of Baltimore, died at his home in that city, Saturday, December 7, 1895, of chronic nephritis, aged forty-seven years. He was the eldest son of the late Jacob J. Michael, and was born and raised on his father's farm in Harford County, Md., on the borders of Chesapeake Bay. In early life he cultivated a taste for water sports and rural surroundings. His preliminary education was received at St. Timothy's Hall, Md., and Newark Academy, Del., and he graduated from Princeton College in 1871. At Princeton he was distinguished for skill in athletic exercises and at graduation was a splendid specimen of physical manhood. He received his doctorate degree from the University of Maryland in March, 1873, and after graduation spent a year abroad in study in the hospitals and medical schools of Europe.

J. EDWIN MICHAEL, M. D.

In the autumn of 1874, Dr. Michael was appointed demonstrator of anatomy in the University of Maryland; in 1880 he was promoted to the chair of anatomy and clinical surgery; in 1887 he was elected dean of the faculty of the University, and in 1890 was chosen professor of obstetrics, which chair he held until his death. For a short time he edited the *Maryland Medical Journal*, and at the time of his death was president of the Medical and Chirurgical Faculty of Maryland; fellow of the American Association of Obstetricians and Gynecologists; member of the American Surgical Association; of the Southern Surgical and Gynecological Association; of the American Medical Association, and of the various local medical societies in Baltimore.

Dr. Michael married Miss Susie Mitchell, of Harford County, December, 1875. His widow and six children, four sons and two daughters, survive him. Dr. Michael was a man of striking physical, intellectual and personal characteristics. He was a man of broad culture, great strength of character, an excellent teacher and a famous physician. He died in the midst of a useful professional life, mourned by his kindred and a large circle of devoted friends.

For the excellent picture that accompanies this sketch we are indebted to the courtesy of the *Maryland Medical Journal* and have condensed the foregoing remarks from an editorial in that *Journal* of December 14, 1895.

## Figures and Tables

**Figure f1:**